# Is the clinician an independent variable in embryo transfer outcomes under standardized direct and indirect supervision? A 5-year observational cohort study

**DOI:** 10.3205/zma001215

**Published:** 2019-02-15

**Authors:** Monica Mittal, Prasanna Raj Supramaniam, Lee Nai Lim, Haitham Hamoda, Mike Savvas, Nitish Narvekar

**Affiliations:** 1 Oxford University Hospitals NHS Foundation Trust, John Radcliffe Hospital, Oxford, UK; 2 King’s College Hospital NHS Foundation Trust, Denmark Hill, Brixton, UK

**Keywords:** embryo transfer, learning curves, assisted reproductive treatment, learning theories, pregnancy rates

## Abstract

**Objective: **To compare the cumulative pregnancy rate (CPR) for experienced clinicians and trainees naive to the skill of embryo transfer (ET) during an assisted reproductive treatment (ART) cycle. To establish the minimum number of procedures required to achieve consistent outcomes.

**Method:** A non-interventional retrospective observational cohort study looking at all consecutive ETs undertaken over a 5-year study period. The CPR was determined by a self-reported urinary home pregnancy test undertaken 16 days after oocyte retrieval.

**Results:** The CPR did not differ between an experienced clinician (39%) and trainee (45%) for the first 50 (p=0.41) and last 50 (40.7% versus 42.7%) (p=0.81) ET procedures. The CPR for the individuals remained consistent with their peaks and troughs mirroring the overall success rate of the unit. This pattern continued when the data was further stratified for co-variables (age [≤37 years of age], catheter type [soft] and embryo quality [expanded blastocyst of grade ≥2]): CPRs for experienced clinicians was 65.7% (first 50 transfers) and 40.9% (last 50 transfers); CPR for trainees was 66.7% (first 50 transfers) and 53.6% (last 50 transfers); p=0.95 and p=0.37, respectively. The trainees, however, were more likely to use a stylet catheter with a 2-step transfer technique, with a cost over clinical implication. Furthermore, patients expressed a preference for an experienced clinician to perform their procedure, despite being informed that the grade of the clinician had no impact on the cycle outcome after an analysis of the unit’s data.

**Conclusion:** The clinician's grade and duration of service have not been shown to significantly impact the outcome of the ART cycle. The findings, however, should be interpreted with caution, as they reflect the culture of training in the unit, where there is a strong emphasis on adequate direct and indirect supervision. Furthermore, the relationship between the volume of work and outcomes is established in postgraduate medical education, with the exact number required to achieve clinical competence being dependent on the procedure and intensity of the workload.

## Zusammenfassung

**Hintergrund: **Embryotransfer (ET) ist die letzte kritische Phase innerhalb des assistierten reproduktiven Behandlungszyklus (ART). Es wird zunehmend deutlich, dass ein atraumatisches Verfahren wichtig für das Erzielen eines erfolgreichen Ergebnisses ist, und somit sollten theoretisch die Erfolgsraten zwischen Kliniksärzten variieren, basierend auf ihrer Position und ihrem Dienstalter, die beide Maßeinheiten klinischer Erfahrung sind. Diese Studie vergleicht die kumulative Schwangerschaftsrate (CPR) erfahrener Kliniker mit der von Ärzten in der Ausbildung, die mit den Fertigkeiten des ET nicht vertraut sind.

**Methode: **Nicht-interventionelle retrospektive beobachtende Kohortenstudie, die über einen 5-Jahres-Zeitraum alle durchgeführten konsekutiven ETs untersucht. Die CPR wurde durch einen Urinschwangerschaftstest festgestellt, der 16 Tage nach der Gewinnung der Oozyte durchgeführt wurde.

**Ergebnisse: **Die CPR zeigte keine Unterschiede zwischen erfahrenen Kliniksärzten (39%) und Ärzten in der Ausbildung (45%) bei den ersten 50 (p=0,41) und den letzten 50 Verfahren (40,7% versus 42,7%) (p=0,81). Die CPR blieb für diese Personen konsistent hinsichtlich Spitzen und Tiefpunkten, die die Erfolgsrate der Abteilung widerspiegelten. Dieses Muster setzte sich fort, als nach Co-Variablen stratifiziert wurde (Alter [≤37 Jahre], Kathetertyp [weich] und Embryoqualität [ausgedehnte Blastozysten von Grad ≥2]): die CPR für erfahrene Kliniker lag bei 65,7% (erste 50 Transfers) und 40,9% (letzte 50 Transfers); die CPR für Ärzte in der Ausbildung lag bei 66,7% bzw. 53,6%; p=0,95 bzw. p=0,37. Ärzte in der Ausbildung neigten eher dazu, einen Mandrinkatheter mit 2-stufiger Transfertechnik zu verwenden mit klinischen Auswirkungen bzgl. Mehrkosten. Darüber hinaus präferierten die Patienten, das ein erfahrener Kliniker bei ihnen das Verfahren durchführen sollte, obwohl sie darüber informiert worden waren, dass nach Analyse der Daten die Dienstposition keinen Einfluss auf das Zyklusergebnis hat.

**Schlussfolgerung: **Dienstliche Position und Dienstalter wirkten sich nicht signifikant auf das Ergebnis des ART-Zyklus aus. Die Ergebnisse sollten jedoch mit Vorsicht interpretiert werden, da sie die Ausbildungskultur in der Abteilung reflektieren, wo großer Wert auf adäquate direkte und indirekte Supervision gelegt wird. Außerdem wird das Verhältnis zwischen Arbeitsvolumen und Ergebnissen in der postgraduierten ärztlichen Ausbildung mit einer genauen Anzahl festgelegt, die nötig ist, um klinische Kompetenz zu erreichen in Abhängigkeit von Verfahren und Intensität des Arbeitsaufwands.

## Background

Embryo transfer (ET) is the final critical stage of an in vitro fertilization (IVF) treatment cycle. It is increasingly clear that a soft atraumatic transfer of embryos into the centre of the uterine cavity, is important to achieve a successful outcome [1], and therefore, in theory, success rates should vary between clinicians based on their grade and length of service, both of which are measures of clinical experience [2].

Training programmes have been developed to support both the theoretical and practical components of the embryo transfer procedure [https://britishfertilitysociety.org.uk/education-training/embryo-transfer-iui last accessed 2^nd^ April 2017]. A study by Papageorgiou et al. (2001) [3], looked at the training of providers in ET. They found that the pregnancy rates were lowest for the first 25 ETs performed by trainees, but were equivalent to experienced clinicians by 40-50 transfers.

To test this hypothesis, we reviewed the cumulative pregnancy rates of consultants and trainees who had worked for a minimum of 2 years during the study period. Whilst the consultants had been fully trained and accredited to perform ETs, the trainees were naïve to the skill, and started performing ET procedures within 6 months of joining the unit. The data was collected retrospectively for their first and last 50 consecutive ETs, and further stratified for variables known to impact the outcome of an assisted reproductive treatment cycle.

## Materials and Method

This is a non-interventional retrospective observational cohort study comparing the pregnancy rates for three experienced consultant clinicians with three trainee fellows. No approval from the institutional review board was deemed necessary in view of the retrospective non-interventional nature of the study design. The study was conducted under standardised conditions required to perform a routine ET procedure. The data was collected for each individual clinician’s first 50 (Group A) and last 50 (Group B) consecutive ETs performed in a single fertility unit undertaking both NHS and self-funded cycles, from the unit computerised database maintained by the embryology team. Each trainee fellow had been in the post for a minimum of two years prior to data regarding their last 50 ETs were collected from. The data was further subdivided to account for confounding variables known to impact cycle outcomes: age of the patient; number of embryos transferred; stage of ET; and, type of catheter used. The data was collected over a period of 5 years to account for post commencement times.

A positive cycle outcome was documented following a self-performed urinary home pregnancy test, 16 days after oocyte retrieval; biochemical pregnancy rates were considered more reflective of the ET outcome in this study, to eliminate bias created by early pregnancy losses which may be secondary to a number of other factors. The overall pregnancy rate refers to the cumulative biochemical pregnancy rate. 

All trainees had been trained in the skill of ET by one or more of the experienced consultant clinicians in the same unit, prior to being allowed to perform ETs independently. The competency framework within the unit indicated a minimum of 20 ETs to be observed prior to being able to perform them under direct supervision. A further 20 ETs were then performed under direct supervision after which the trainee had to be deemed competent to perform them under indirect supervision. Competency was then further reassessed after a minimum of 20 ETs prior to being able to perform them independently. All transfers were performed using a Wallace catheter (either size 18 or 23 with or without a stylet introducer) with the patient in lithotomy position under transabdominal ultrasound guidance for correct catheter placement.

The number 50 was based on the minimum number of transfers required by the British Fertility Society Embryo Transfer Training Module. This is further supported by other reports of training experiences [3], [4], [5], [6].

A prospective qualitative assessment of the clients understanding and wishes regarding their ET procedure was undertaken. This was performed through a questionnaire which they completed immediately prior to their transfer. The questionnaire was divided into Part A and Part B. Part A obtained: baseline information regarding the type of cycle being undertaken; enquired as to the patient’s thoughts as to what contributes to the outcome of their cycle; and, whom they would prefer to carry out their ET procedure. In Part B, the patient was advised that a detailed analysis of the last 3 years of ET outcomes in the unit was not influenced by the grade of the clinician undertaking the procedure. They were then asked to indicate whom they would like to undertake their procedure.

## Results

The mean (±standard deviation [SD]) age of the patients included within Group A performed across all day transfers was 34.76 (±4.07) years (range 23 to 42). When this is further subdivided into the age for all first cleavage stage transfers and blastocyst stage transfers, the mean age was 35.41 (±3.73) years and 33.75 (±4.30) years, respectively. The mean (±SD) age of all patients included within Group B performed across all day transfers was 34.90 (±4.17) years (range 23 to 44 years). When this is further subdivided into the age for all cleavage stage transfers and blastocyst stage transfers, the mean age was 36.52 (±3.38) years and 33.58 (±4.30) years, respectively. Table 1 (Tab. 1) summaries the clinical pregnancy rate and characteristics of the first 50 ETs.

In Group A, experienced consultant clinicians performed 72 ETs (48.0%) in women less than 35 years of age, and trainee clinicians performed 76 ETs (50.7%) in women less than 35 years of age, (p=1.00). In Group B, experienced consultant clinicians performed 74 ETs (49.33%) in women less than 35 years of age, and trainee clinicians performed 79 ETs (52.67%) in women less than 35 years of age, (p=0.56).

In Group A, a total of 134 (44.7%) ETs were performed using a soft catheter, with experienced clinicians opting for a soft catheter in 53.3% of transfers compared with trainee clinicians opting for a soft catheter in 36.0% of their procedures, (p<0.05). In Group B, a total of 148 (56.9%) ETs were performed using a soft catheter, with experienced clinicians opting for a soft catheter in 61.3% of transfers compared with trainee clinicians opting for a soft catheter in 53.7% of their procedures, (p<0.05).

In Group A, experienced clinicians opted for a soft catheter in 58.6% of blastocyst transfer procedures compared to trainee clinicians who opted for a soft catheter in 28.6% of blastocyst transfers, (p<0.05). In Group B, experienced clinicians opted for a soft catheter in 58.2% of blastocyst stage transfer procedures compared to trainee clinicians who opted for a soft catheter in 50% of blastocyst stage transfers, (p<0.05).

**Figure 1:**
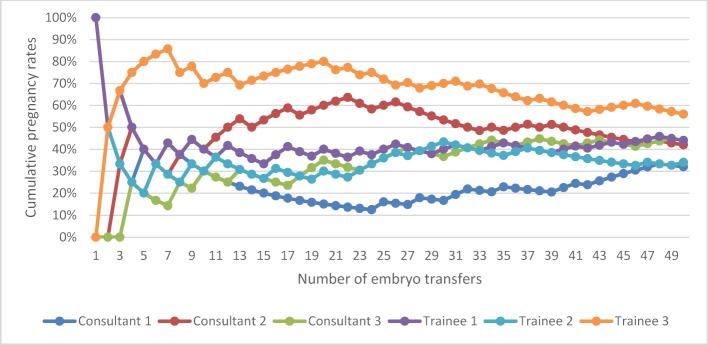
Cumulative pregnancy rates learning curve for the first 50 embryo transfers performed by an experienced clinician (consultant) and trainee (fellow)

The overall pregnancy rate for Group A, for all grades of clinicians, both experienced and trainees, was 42.0%. The pregnancy rate for experienced clinicians was 39.3% and 44.7% for trainees, (p=0.35). The pregnancy rate for women less than or equal to 35 years of age was 45.9%, and 38.2% for women greater than 35 years of age, (p=0.18). Figure 1 (Fig. 1) illustrates the learning curves for the first 50 ETs performed by three experienced clinicians (consultants) and the three trainees (fellows). The individual learning curves vary with a similar end-point seen after 50 transfer procedures and a plateauing effect demonstrated after approximately 12 procedures have been undertaken. Experience does not reflect a direct proportional correlation with success, demonstrated by Fellow 3 having higher pregnancy rates compared to Consultant 1.

In Group A, the pregnancy rate for cleavage stage transfers was 34.4% and 56.5% for blastocyst stage transfers, (p<0.05). Experienced clinicians had a pregnancy rate of 51.7% for blastocyst stage transfers and trainees had a pregnancy rate of 58.9%, (p=0.53).

**Figure 2:**
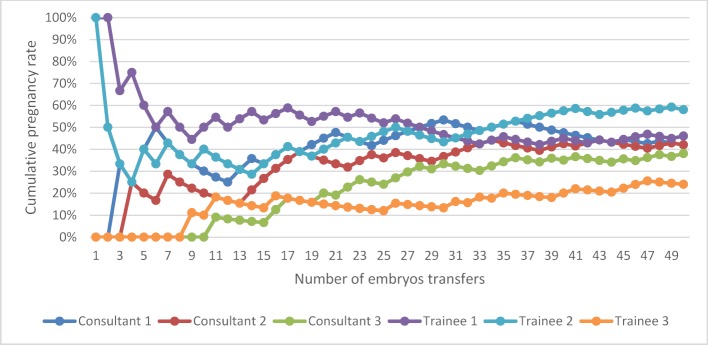
Cumulative pregnancy rates learning curve for the last 50 embryo transfers performed by an experienced clinician (consultant) and trainee (fellow)

The overall pregnancy rate for Group B, for all grades of clinicians, both experienced and trainees, was 41.7%. The pregnancy rate for experienced clinicians was 40.7% and 42.7% for trainees, (p=0.73). The pregnancy rate for women less than or equal to 35 years of age was 52.9%, and 29.9% for women greater than 35 years of age, (p<0.05). Table 2 (Tab. 2) and figure 2 (Fig. 2) illustrates the cumulative pregnancy rates learning curves for the last 50 ETs performed by the same three experienced clinicians and three trainees. With time, and experience, the pregnancy rates for the individual clinicians show a more consistent outcome, with no peaks or troughs, and Fellow 3, now having the lowest pregnancy rates despite consistency in practice and procedures.

In Group B, the pregnancy rate for cleavage stage transfers was 26.9% and 52.4% for blastocyst stage transfers, (p<0.05). Experienced clinicians had a pregnancy rate of 52.1% for blastocyst stage transfers and trainees had a pregnancy rate of 52.7%, (p=0.93). 

**Figure 3:**
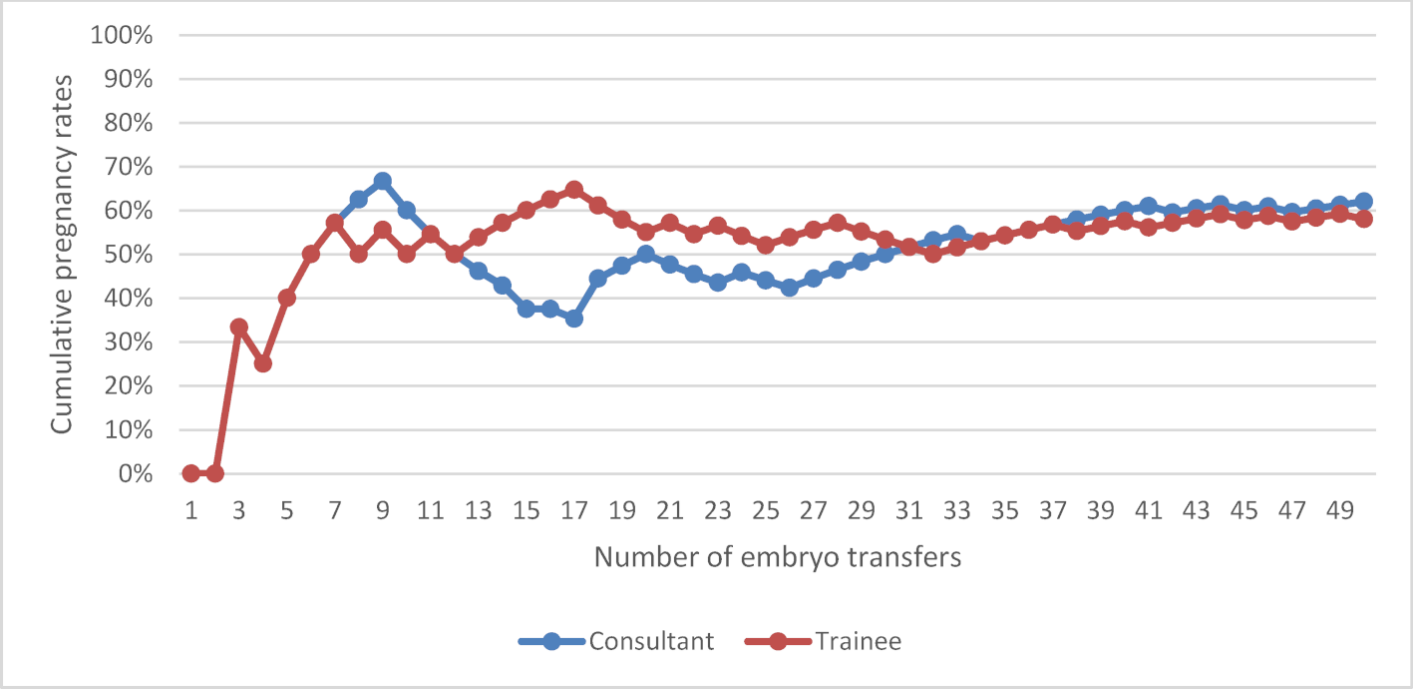
Cumulative pregnancy rates learning curve for the first 50 embryo transfers stratified for by potential covariables, performed by an experienced clinician (consultant) and trainee (fellow)

**Figure 4:**
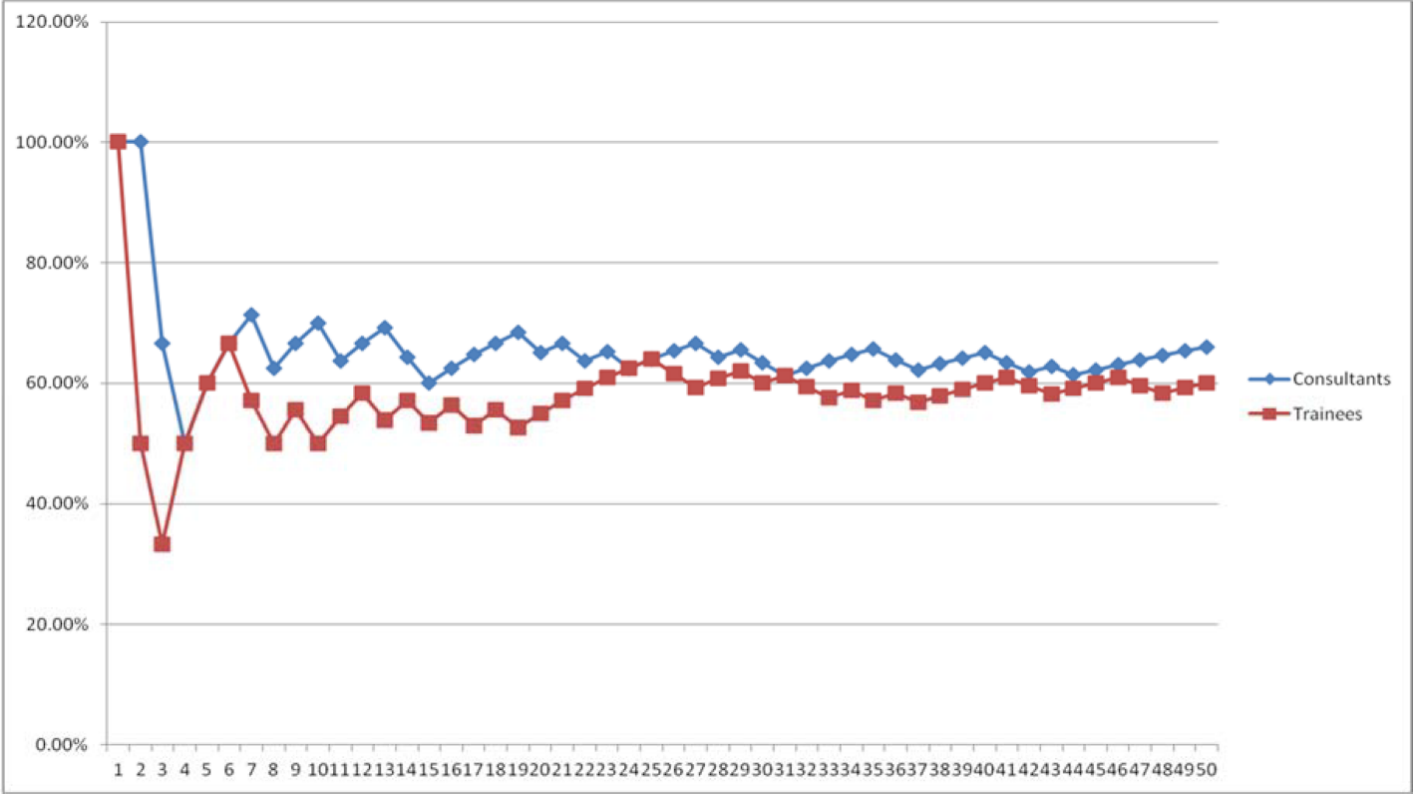
Cumulative pregnancy rates learning curve for the last 50 embryo transfers stratified for by potential covariables, performed by an experienced clinician (consultant) and trainee (fellow)

The data has been further analysed to control for co-variables thought to impact the outcome of the cycle (see table 3 (Tab. 3) and table 4 (Tab. 4)), including: age (≤37 years of age); catheter type (soft); and, embryo quality (expanded blastocyst of grade ≥2), for the first and last 50 ETs performed by experienced consultant clinicians and trainees. The overall pregnancy rate for: experienced clinicians when accounted for the confounding variables was 65.7% and 40.9%, respectively for the first and last 50 ETs; and 66.7% and 53.6% for trainees. No significant difference is noted in the learning curves for the two broad groups of practicing clinicians (see figure 3 (Fig. 3) and figure 4 (Fig. 4)): p=0.95 for the first 50 transfer procedures; and, p=0.37 for the last 50 transfer procedures.

Twenty-four questionnaires were returned from patients prospectively asked about the ET procedure that they were due to undergo. 54.2% strongly felt that the quality of the embryo transferred had an impact on the outcome of their treatment cycle. 41.7% felt that the grade of the clinician performing the procedure had an impact on the outcome of their treatment with 54.2% preferring an experienced consultant to perform their procedure. After, being informed that a detailed analysis of the last 3 years of ET outcomes in the clinic indicated that the chance of a successful pregnancy is not influenced by the experience or grade of the doctor performing the transfer, 16.7% of questioned clients changed their mind with now 50.0% opting for an experienced clinician.

## Discussion

Embryo transfer (ET) is one of the last steps within an assisted reproductive treatment cycle and is considered to be crucial in predicting the outcome. The grade of the embryo, the difficulty of the procedure and endometrial receptivity are all accepted contributory factors of the ET result [3].

Analysis of the data from a single fertility unit undertaking both NHS and self-funded cycles has demonstrated that one variable, the experience and grade of the clinician undertaking the procedure, previously considered to have an impact on the outcome of the assisted reproductive treatment cycle, may no longer significantly affect the outcome, given the right training environment and exposure.

In this study, the mean age of the women in both Group A and Group B was 35 years of age with similar aged women undergoing cleavage stage and blastocyst stage transfers, with no statistically significant difference in the number of transfers performed in women under the age of 35 years by either grade of clinician, (experienced or trainee). However, an experienced clinician was more likely to opt for a soft catheter than a trainee (p<0.05), with an increasing level of confidence seen over time in trainees with a greater proportion of the transfers performed by them using a soft catheter in the last 50 ET procedures undertaken. This difference had no significant impact on the overall pregnancy rate for the unit and for both grades of clinicians over their first and last 50 transfer procedures. A significantly greater success rate was documented for women less than 35 years of age in the last 50 ET procedures. The pregnancy rate was also significantly greater for blastocyst stage transfers compared with cleavage stage embryos. As women, less than 35 years of age and a blastocyst stage transfer are both positive correlations to a pregnancy rate, this would explain the greater success in this group independent of the clinician. Experienced and trainee clinicians had equivalent success rates for blastocyst stage transfers for both their first and last 50 transfer procedures.

Further analysis of the data, stratifying for the co-variables, age, quality of embryo and type of catheter used, known to impact the outcome of assisted reproductive treatment cycles, showed no significant difference for pregnancy rates between ET procedures performed by an experienced clinician or a trainee.

Individual clinicians have been shown to have a varied acquisition of competence with pregnancy rates stabilising after the first 12 transfers performed, in contrast to the study by Segars (2001) [7] who suggested that after 50 transfers the rate is comparable. 

The patient questionnaire demonstrated that their overall perception regarding the impact of embryo quality on cycle outcome was in keeping with current scientific evidence and practice. However, they expressed a preference for an experienced clinician to perform their procedure despite less than 50% believing that the clinician had an impact on their cycle outcome. This preference did not change despite being informed that the grade of the clinician had no impact on the cycle outcome after an analysis of the unit’s data.

Kovacs (1999) [8], distributed a questionnaire to clinicians in Australia and New Zealand to determine their attitude to 12 factors thought to impact the outcome of an assisted reproductive treatment cycle. The clinicians felt that the presence of a hydrosalpinx had the greatest impact followed by the difficulty of the procedure depicted by the presence of blood on the catheter and the use of a tenaculum. The clinicians however, were not asked to rate the impact that a physician has on the outcome of the treatment cycle.

Another postal survey of ET practice in the UK [3], looking at the attitudes of clinicians to factors that may influence the ET procedure, found that most clinicians felt that a standardised protocol was needed and that the presence of blood on the catheter tip were the most influential factors. The need for bed rest was considered the least significant. Again, the clinician undertaking the ET were not asked to rate their impact on the treatment cycle.

Naaktgeboren et al., (1997) [9] highlighted differences in practices and success rates between clinicians working within the same unit. Some studies have reported large differences in the ongoing pregnancy rates between various physicians working within the same unit, ranging between 17.0% to 54.0% in one study and 13.2% to 37.4% in another study [2], [10]. The pregnancy rate range for the 6 clinicians in this study for the last 50 ETs performed including both cleavage and blastocyst stage embryos was 24.0% to 58.0%. When subdivided to account for other confounding variables that can impact the outcome of a cycle, quality of embryo replaced, the rate ranged between 42.0% to 66.0%. Further analysis to stratify for age, quality of embryo and type of catheter used, narrowed the pregnancy rate range even further, from 60.0% to 66.0%. Standardisation of the ET protocol has been shown to reduce the differences in the success rate of the various clinicians [9]. Controlling for co-variables has been shown to limit the range further. However, not all variables can be accounted for, and this can contribute to the differences seen. Overall, the findings of this study showed no statistical difference in pregnancy rate for experienced clinicians compared with trainees, supporting the findings by van Weering HGI et al., (2005) [11] who demonstrated no statistically significant difference in success rates between six physicians undertaking ETs within the same unit. 

The data presented in this study has also shown varying results to previous studies with regards to catheter type. No significant difference was found in pregnancy success rates for transfers performed using a soft catheter or a firm catheter with a stylet: first 50 ET procedures, p=0.38 (soft, 44.8%; firm, 39.8%); last 50 ETs, p=0.66 (soft, 43.2%; firm, 38.4%). The study by Kovacs (1999) [8] and Papageorgiou et al., (2001) [3] found that the clinicians questioned ranked the type of catheter as the third and fourth most significant factor in the ET procedure, respectively. However, a systematic review and meta-analysis performed by Abou-Setta AM et al., (2005) [1] concluded that an ET performed using a soft catheter yielded significantly higher pregnancy rates than a firm catheter, secondary to the reduced trauma on the endometrium and reduced rate of uterine contractility. They also concluded that the use of a soft catheter can be associated with an increased incidence of traumatic transfers due to the difficulty that could be encountered by trying to negotiate the cervical canal with a soft catheter, but felt that this does not negate from a soft catheter being associated with better pregnancy rates.

The relationship between volume of clinical work and outcomes was established early on in medical education. It has also been demonstrated that a set amount of time is taken for an individual to reach an end goal [12]. The concept of quantitatively assessing proficiency in clinical skills for individuals has gathered prominence particularly in postgraduate medical education following on from learning points raised by previous mortality and morbidity reports. The number of procedures required to reach clinical competence has always been a topic of debate, historically based on subjective criteria, including number of attempts and time since training. However, it should be noted that an arbitrary set number of attempts does not take into account inter-individual variability in learning, nor does it provide for continued assessment of maintained proficiency over time [13]. This does not preclude from setting a minimum number of procedures required to be performed over a set period of time in order to maintain proficiency, as has been clearly documented to reduce the complication rates in other procedure related environments.

However, in this article we have addressed the number required for this particular skill set. Activity and goal orientated training play a key role in medical education, particularly in a postgraduate environment where success of procedures is evaluated based on clinical outcome. Trainers should actively be aware of these learning theories and use them to enhance the teaching environment, helping to improve outcomes for trainees and clients alike.

This article further highlights the use of Ericsson’s deliberate and repetitive practice theory. Although not directly thought off in the medical education concept, trainers have for long used this educational principle to develop surgical skills in new trainees. Whilst, in Ericssons theory [14], the effect of natural ability on the development of an expert skill was explored, in this study we have highlighted that there was no significant variation between trainees at the beginning, in terms of cumulative pregnancy rate or the index number of procedures at which they achieved clinical competence. One would expect, that should the natural ability have an impact this variation would be observed. Trainees were also encouraged to utilise the Kolb’s model of experiential learning [15], where they reflected on the success and failures of ET’s performed, whereby this experience formed an active experimentation base that they built their overall concrete experience in performing ETs on.

## Conclusions

The data shows, that both the grade of the clinician and duration of service does not have a significant impact on the outcome of the assisted reproductive treatment cycle. Other variables known to impact the outcome, hold greater predictive value. The findings, however, should be interpreted with caution, as they reflect the culture of training within this unit, where there is a strong emphasis on adequate direct and indirect supervision of early ETs, potentially being a cause for bias in the outcome seen. Furthermore, ETs that were difficult or anticipated to be difficult were more likely to be carried out by an experienced clinician, and this should be considered when interpreting the data.

## Declarations

### Ethics approval and consent to participate

Ethical approval was not deemed necessary as this was a non-interventional study.

#### Availability of data and materials

The datasets generated and/or analysed during the current study are not publicly available due to the containment of patient identifiable information but are available from the corresponding author on reasonable request.

#### Authors’ contributions

MM collected the data. MM and PRS analysed the data. MM, PRS, LL, HH and MS helped formulate the manuscript. NN oversaw the whole process. All authors read and approved the final manuscript.

## Competing interests

The authors declare that they have no competing interests.

## Figures and Tables

**Table 1:**
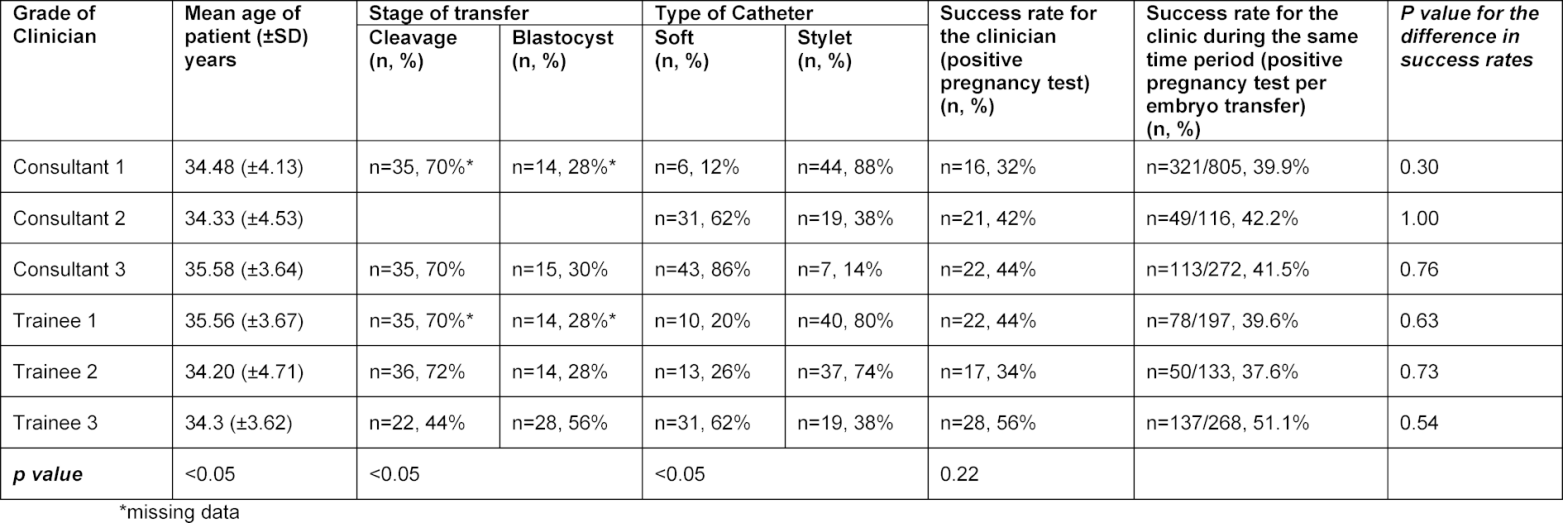
Clinical pregnancy rates and characteristics of the first 50 embryo transfers performed

**Table 2:**
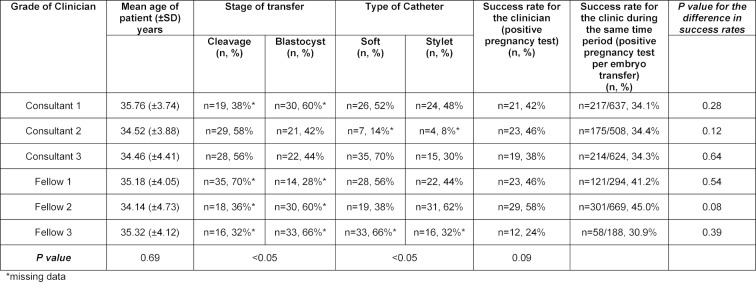
Clinical pregnancy rates and characteristics of the last 50 embryo transfers performed

**Table 3:**
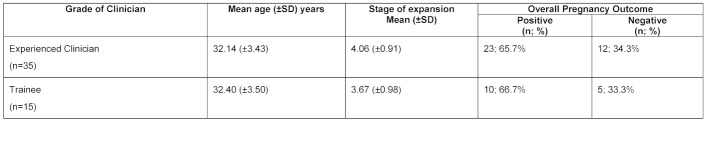
Clinical pregnancy rates and characteristics of the first 50 embryo transfers performed stratifying for covariables

**Table 4:**
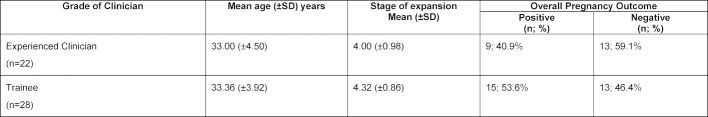
Clinical pregnancy rates and characteristics of the last 50 embryo transfers performed stratifying for covariables
